# Network-Based Approach to Identify the Antiproliferative Mechanisms of Bruceine D in Breast Cancer From the Cancer Genome Atlas

**DOI:** 10.3389/fonc.2020.01001

**Published:** 2020-07-02

**Authors:** Saisai Tian, Rui Jing, Weidong Zhang

**Affiliations:** ^1^School of Pharmacy, Second Military Medical University, Shanghai, China; ^2^Institute of Interdisciplinary Integrative Medicine Research, Shanghai University of Traditional Chinese Medicine, Shanghai, China

**Keywords:** Bruceine D, breast cancer, bioinformatics, WGCNA, GSNCA

## Abstract

Bruceine D (BD) is a natural compound extracted from a Chinese herb *Brucea javanica* that has been used for anti-inflammatory and anti-cancer treatment. However, little is reported about BD's effects in breast cancer tumorigenesis. In this paper, we aimed to investigate the effect of BD in breast cancer and elucidate the potential mechanism of BD by integrated multiple databases. Our data suggested BD inhibited MCF-7 and MDA-MB-231 cells proliferation and promoted cells apoptosis. We integrated multiple bioinformatics analysis strategies to identify genes, hub modules and pathways associated with BD treatment. Three key pathways, including AMIT_SERUM_RESPONSE_40_MCF10A, BILD_HRAS_ONCOGENIC_SIGNATURE, and NAGASHIMA_NRG1_SIGNALING_UP were identified to be associated with therapeutic effects of BD in breast cancer. Additionally, we validated the key genes by using quantitative real-time PCR and western blot. In conclusion, these findings revealed potential molecular mechanisms of BD to treat breast cancer by affecting AMIT_SERUM_RESPONSE_40_MCF10A, BILD_HRAS_ONCOGENIC_SIGNATURE, and NAGASHIMA_NRG1_SIGNALING_UP pathways and regulating expression of ZFP36, EGR1, and FOS.

## Introduction

Breast cancer is one of the most common malignancies with high rates of morbidity and mortality in women (1–4). Over the past 20 years, the incidence rate of breast cancer is increasing in China (5–7). Although, several therapeutic strategies, including radiation therapy, chemotherapy, surgery and molecule-targeted therapy, improve survival rates greatly, many patients still face recurrence, drug resistance and serious side effects (8–11). Therefore, it is urgent to identify a more effective therapy.

Recently, natural compounds have been considered as an important source of anti-tumor drugs (12, 13). Bruceine D (BD) is a natural compound derived from a herb of *Brucea javanica*. It has been reported that BD has good pharmacological activities including cytotoxic effects on cancer cell, anti-inflammatory and hypoglycemic activities (2, 14–17). However, little is reported about BD's effects in breast cancer. In our present study, we used breast cancer cells MCF-7 and MDA-MB-231 to test the effects of BD. Results illustrated that the intervention with BD significantly inhibits the proliferation and induces apoptosis of MCF-7 and MDA-MB-231 cells. In recent years, integrated bioinformatics analysis has been applied to understand molecular mechanisms of cancer and drug action mechanism (18, 19). A large amount of genomic data was generated which has aided cancer studies considerably. Thus, we investigate the underlying pharmacological mechanism of BD in breast cancer by integrated bioinformatics analysis. We identified three pathways, including AMIT_SERUM_RESPONSE_40_MCF10A BILD_HRAS_ONCOGENIC_SIGNATURE and NAGASHIMA_NRG1_SIGNALING_UP pathways and three genes, including ZFP36, EGR1, and FOS. Finally, we adopt quantitative real-time PCR and western blot to validate the effects of BD on ZFP36, EGR1 and FOS expression in MCF-7 and MDA-MB-231 cells.

In this work, we firstly reported that BD significantly inhibited MCF-7 and MDA-MB-231 breast cancer cells proliferation and induced S phase cell cycle arrest and apoptosis in MCF-7 and MDA-MB-231 cells. Integrated computational analysis results indicated that BD mainly affects AMIT_SERUM_RESPONSE_40_MCF10A, BILD_HRAS_ONCOGENIC_SIGNATURE, and NAGASHIMA_NRG1_SIGNALING_UP pathways and regulates expression of ZFP36, EGR1, and FOS.

## Materials and Methods

### Chemical and Reagents

Bruceine D was purchased from Shanghai Zaiqi Bio-Tech (Shanghai, China). Cell counting kit-8 (CCK-8) was obtained from Dojindo Laboratories (Kumamoto, Japan). The cell cycle kit was purchased from MultiSciences (Lianke) Biotech Co., Ltd (Hangzhou, China), and the Annexin-V/propidium iodide (PI) double-labeled flow cytometry kit was obtained from KeyGen Biotech Co., Ltd (Nanjing, China). Goat anti-rabbit horseradish peroxidase (HRP)–conjugated secondary antibodies was acquired from Cell Signaling Technology (USA). Rabbit anti- ZFP36 (catalog no. abw8) and anti-EGR1 (catalog no. ab133695) were from Abcam (UK). Rabbit anti- ZFP36 (catalog no. ab33058) and anti-EGR1 (catalog no. ab133695) were from Abcam (UK). Rabbit anti-FOS (c-FOS) (catalog no. 2250) and anti-GAPDH (catalog no. 5174) were purchased from Cell Signaling Technology (USA).

### Cell Culture

Human breast cells MCF-7 and MDA-MB-231 were cultured in DMEM supplemented with 10% Fetal Bovine Serum (FBS), 100 μg/ml of streptomycin, and 100 U/ml of penicillin in a humidified incubator at 37°C where the carbon dioxide content is 5%. The cells were grown to 70% confluence in monolayer and treated with indicated concentrations of BD for experiment, dissolved in dimethyl sulfoxide and subsequently diluted in cell culture medium.

### Cell Cycle Distribution Assay and Apoptosis Assay

MCF-7 and MDA-MB-231 cells were treated with 0 μM (control), 0.1, 0.5, and 1.5 μM BD for 72 h. According to the instructions of the cell cycle detection kit, the cell cycle distributions were identified by PI staining and followed analyzed by flow cytometric. The BD's effect on cell apoptosis was evaluated using the Annexin-V/PI double-labeled flow cytometry kit. Cells were analyzed by flow cytometry with a FACS Calibur flow cytometer (BD Biosciences, San Jose, CA, USA).

### Data Collection and Preprocessing

The mRNA raw count profiles of the TCGA-BRCA project were downloaded from the GDC data portal (https://portal.gdc.cancer.gov). Total of 1109 BRCA patient samples and 113 control samples were available in TCGA. Simultaneously, the GSE85871 datasets were obtained from the Gene Expression Omnibus database (https://www.ncbi.nlm.nih.gov/geo). Raw CEL files of MCF-7 cells treated with BD (GSM2286384 and GSM2286384) and the respective controls (GSM2286316 and GSM2286317) were downloaded from the GSE85871 dataset which contained the gene expression profiles of 102 TCM ingredients used to treat MCF-7 cells. Raw CEL files were normalized by Robust Multiarray Average using affy package.

### Differential Expression Analysis

Using DESeq2 package, we performed differential expression analysis at the cutoff of |log2 fold change| > 2 and padj < 0.05 (*p*-value adjusted using Benjamini-Hochberg method) in TCGA dataset. For GEO datasets, the limma package was used to screen the differential expression genes at the cutoff of |logFC| > 1 and padj < 0.05 (*p*-value adjusted using Benjamini-Hochberg method). In addition, a pre-ranked gene list of all genes ordered by the absolute value of fold change were constructed in GEO dataset for further analysis.

### Establishment of Gene Co-expression Network

First, we normalized the counts data from TCGA by the TMM algorithm implemented in the edgeR package. Next, the weighted correlation network analysis (WGCNA) was conducted to construct the co-expression network in TCGA dataset using WGCNA package. The detailed procedure for WGCNA construction could be found in our previous study (20). Briefly, according to an appropriate β, we built a weighted adjacency matrix and transformed the adjacency into a topological overlap matrix (TOM). Finally, we performed an average linkage hierarchical clustering according to the TOM-based dissimilarity measure (20). In this study, we chose a minimum module size of 30 for the gene dendrogram and a cut-line (0.25) for the module dendrogram (20).

### Determination of Significant Modules Associated With Breast Cancer

According to gene expression similarities in samples, gene modules were discerned. Simultaneously, using two methods, the correlation was calculated between the gene modules and sample information to find significant modules associated with breast cancer. First, we calculated the module eigengene (ME) of a module to represent the overall expression level of the module. Subsequently, we also calculated gene significance (GS) between gene expression and a sample trait, which was defined as the log10 transformation of the *P*-value (GS = lgP) in the linear regression (20). Finally, the gene module most correlated with sample traits were identified as a module of interest.

### Differential Co-expression Analysis and Gene Set Enrichment Analysis

The gene set net correlations analysis (GSNCA) was conducted to find pathways that were differentially co-expressed between BRCA samples and control samples. There was an assumption that the co-expression network of a pathway did not alter between two conditions in GSNCA, which was implemented in GSAR package. Using the most correlated genes, it builds a core of co-expression network in each condition and finds a hub gene with the highest correlations with the other genes in a pathway. It hinted regulatory changes of the pathway when hub genes in a pathway were different between two conditions. The colors of nodes represent weight factor (w)'s value distributed to all genes for suggesting the mean correlation with any other gene in the gene set co-expression (21). In this paper, it is worth noting that pathways only with 10 ≤ *n* ≤ 500 where n represents the number of genes were kept. Then, using structure of minimum spanning tree-2 (MST2), the most highly correlated pathways were distinguished (21). In this study, the pathway gene sets were used in C2 collection (curated gene sets) from Molecular Signatures Database (MSigDB). In addition, a pre-ranked gene list of all genes ordered by fold change were constructed in BD samples vs. model samples. Then, we performed gene set enrichment analysis (GSEA) for C2 collection (curated gene sets) of MSigDB using clusterProfiler package. Simultaneously, other functional enrichment analysis also was did using clusterProfiler package at a cutoff of *P* < 0.05.

### Quantitative Real-Time PCR Analysis

Human breast cancer cells MCF-7 and MDA-MB-231 were treated with BD at indicated doses for 72 h. The total RNA was extracted from MCF-7 and MDA-MB-231 cells using a commercial kit (TianGen Biotech Co., Ltd, China) according to the manufacturer's instructions, and 500 ng of RNA were reverse transcribed to cDNA using high capacity cDNA reverse transcription kit (TaKaRa Biotechnology Co., Ltd, Japan). The expression of ZFP36, EGR1 and FOS genes were determined by real-time PCR using the ABI Prism 7900 sequence detection system (Applied Biosystems Co., Ltd, USA) and SYBR PrimerScript RT-PCR Kit (TaKaRa Biotechnology Co., Ltd, Japan). The primers were: ZFP36 (Gene bank: 7538) sense, CTGTCACCCTCTGCCTTCTC; anti-sense, TCCCAGGGACTGTACAGAGG, EGR1 (Gene bank:1958) sense, TGACCGCAGAGTCTTTTCCT; anti-sense, TGGGTTGGTCATGCTCACTA and FOS (Gene bank: 2535) sense, AGAATCCGAAGGGAAAGGAA; anti-sense, CTTCTCCTTCAGCAGGTTGG. To control variation in mRNA concentration, all results were normalized to the housekeeping gene, GAPDH.

### Western Blot Analysis

Human breast cancer cells MCF-7 and MDA-MB-231 were grown in 6-wells plates up to 60% confluence, and treated with BD at indicated doses for 72 h. The cells were washed 2 times with phosphate buffered saline (PBS), lysed in 40 μL of cold RIPA lysis buffer containing 1X Phenylmethylsulfonyl Fluoride (PMSF) and then used for western blot assay. Western blot assay was performed as described previously (22). Membranes were probed using rabbit antibodies specific for ZFP36, EGR1, FOS, and GAPDH (1:1000), followed by incubation with HRP-conjugated secondary antibody (1:2000).

### Statistical Analysis

All statistical tests were did using Student's *t*-test or one-way analysis of variance (ANOVA) when appropriate. GraphPad and R software were used for statistical analysis of the original data and the data were expressed as the means ± standard deviation (SD). For all statistic al analysis, a *P* < 0.05 was considered statistically significant.

## Results

### The Effect of BD on MCF-7 and MDA-MB-231 Breast Cancer Cells Proliferation

**Figure 1:**
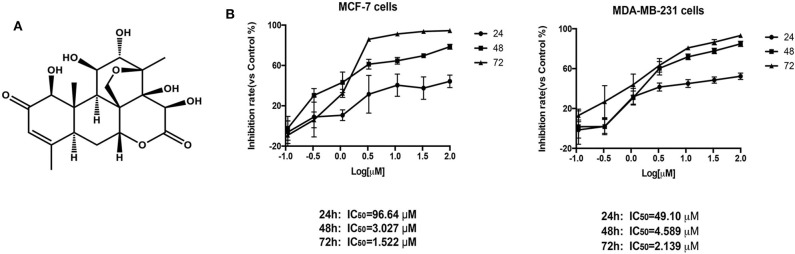
The effect of BD on MCF-7 and MDA-MB-231 breast cancer cells proliferation. **(A)** The chemical structure of BD. **(B)** Cytotoxicity of BD against MCF-7 and MDA-MB-231 breast cancer cells on the CCK-8 assay. Result are mean ± SD (*N* = 3).

After BD treatment of MCF-7 and MDA-MB-231 (triple negative breast cancer) cells at different concentrations for 24, 48, and 72 h, we used CCK8 method to determine the inhibition rates of cell proliferation. As shown in Figure 1, the IC_50_ values of BD at 24, 48, and 72 h were 96.64, 3.027, and 1.522 μM in MCF-7 cells and 49.10, 4.589, and 2.139 μM in MDA-MB-231 cells, respectively. MCF-7 cells showed more sensitivity to BD than MDA-MB-231. These results indicate that the inhibition of BD on MCF-7 and MDA-MB-231 cells proliferation is dose and time dependent.

### The Effects of BD on Cell Cycle and Apoptosis in MCF-7 and MDA-MB-231 Breast Cancer Cells

**Figure 2:**
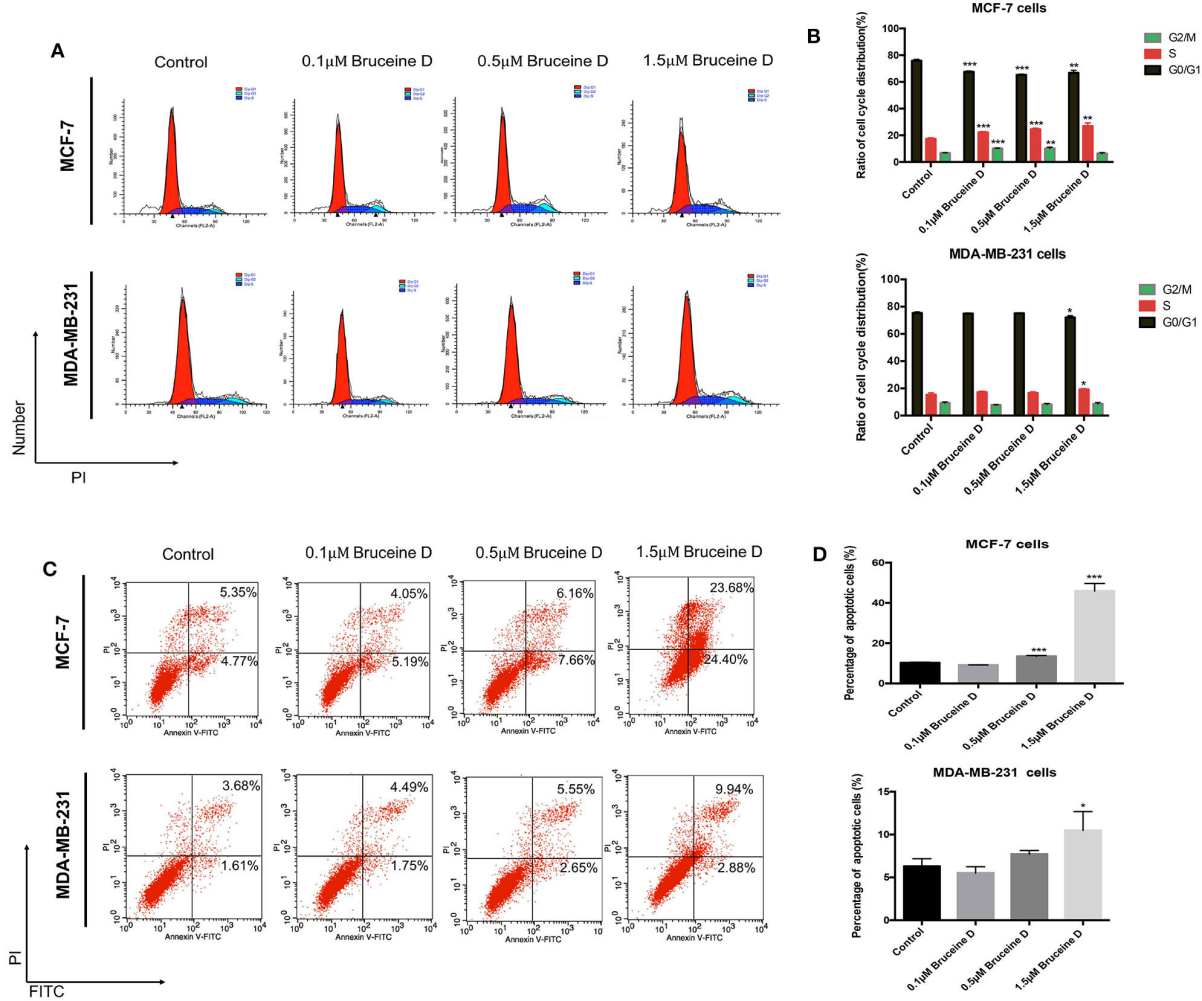
The effect of BD on cell cycle arrest and apoptosis in MCF-7 and MDA-MB-231 cells. **(A,B)** The number of MCF-7 and MDA-MB-231 cells in different stages of cell cycle after treatment with different concentrations of BD for 72 h. **(C,D)** Flow cytometry based on Annexin V/PI double staining to assess extent of apoptosis in two breast cancer lines after treatment with BD for 72 h; *p < 0.05 to control group; **p < 0.01 to control group; ***p < 0.001 to control group (0 μM).

As demonstrated above, BD strongly inhibited MCF-7 and MDA-MB-231 proliferation in a dose-dependent manner. To further explore if BD could induce cell cycle, MCF-7 and MDA-MB-231 cells were treated with 0, 0.1, 0.5, and 1.5 μM of BD for 72 h and then stained with PI for flow cytometry analysis. As shown in Figures 2A,B, the arrest of MCF-7 and MDA-MB-231 cell cycle happened in the S phase and showed BD-concentration dependent. Similar studies for apoptosis were performed using flow cytometry. MCF-7 and MDA-MB-231 breast cells were treated with corresponding concentrations of BD for 72 h and then stained with annexin V/PI. As shown in Figures 2C,D, the ratio of apoptotic cells was clear increased as the concentration of BD increased. These findings indicate that BD could induce S phase cell cycle arrest and apoptosis in MCF-7 and MDA-MB-231 cells in a dose-dependent manner.

### Identification of Modules by Weighted Co-expression Network Analysis

**Figure 3:**
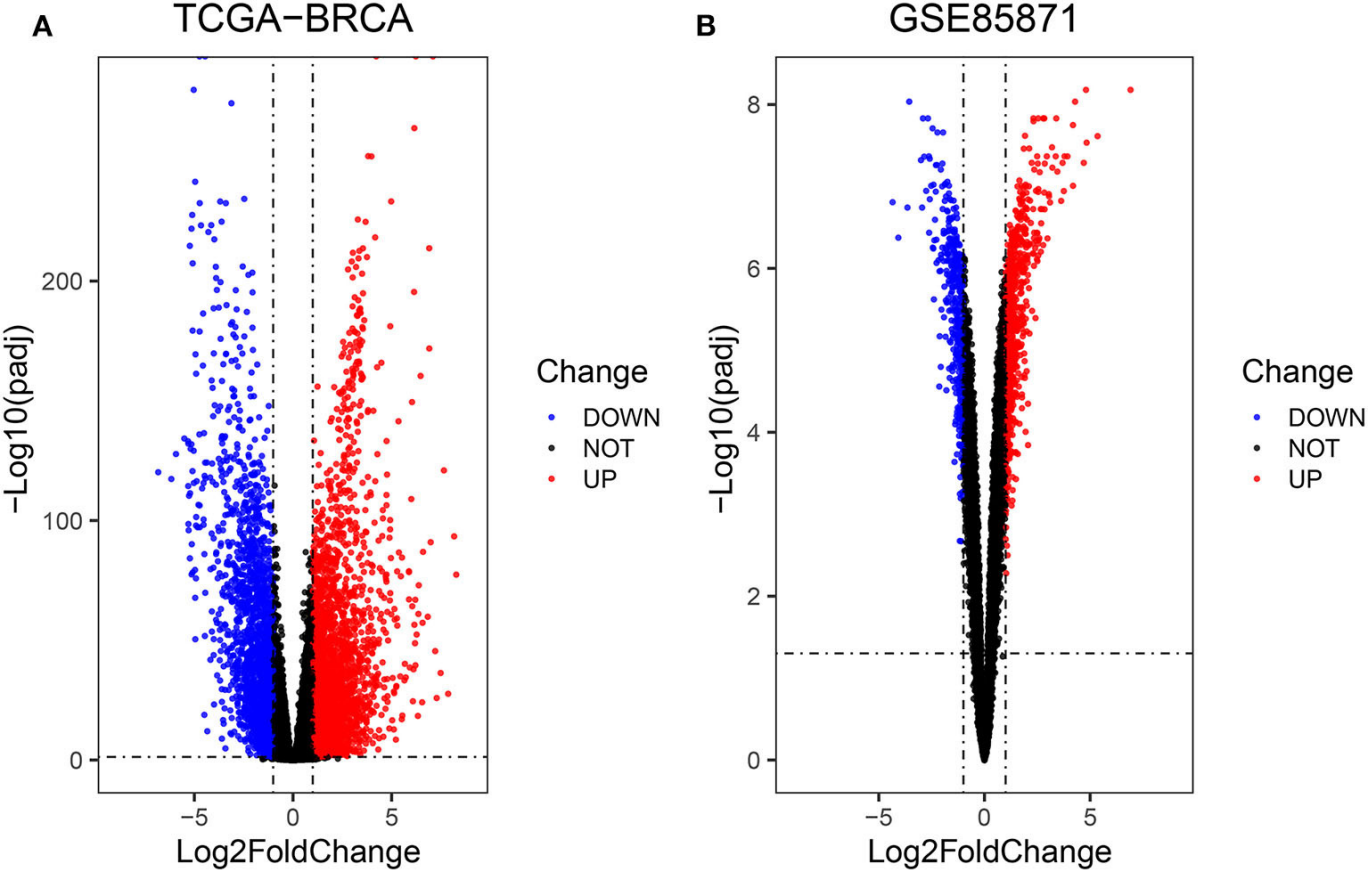
Volcano diagrams of differentially expressed DEGs in TCGA **(A)** and GEO **(B)**. Green dots show downregulated DEGs and red dots show upregulated DEGs.

**Figure 4:**
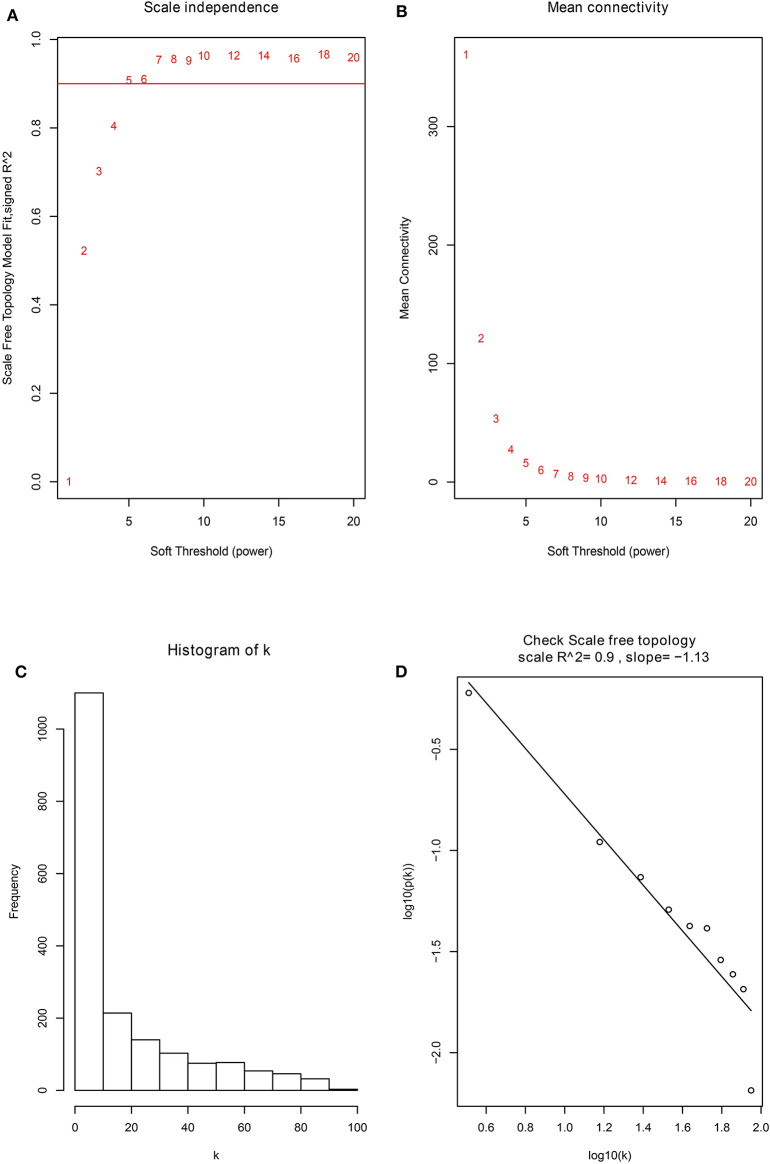
The identification of soft-thresholding power in the weighted gene co-expression network analysis. **(A)** Analysis of the scale-free topology index for various power values between 1 and 20. **(B)** Analysis of the mean connectivity for various power values between 1 and 20. **(C)** Histogram of connectivity distribution when β = 6. **(D)** Checking the scale free topology when β = 6.

**Figure 5:**
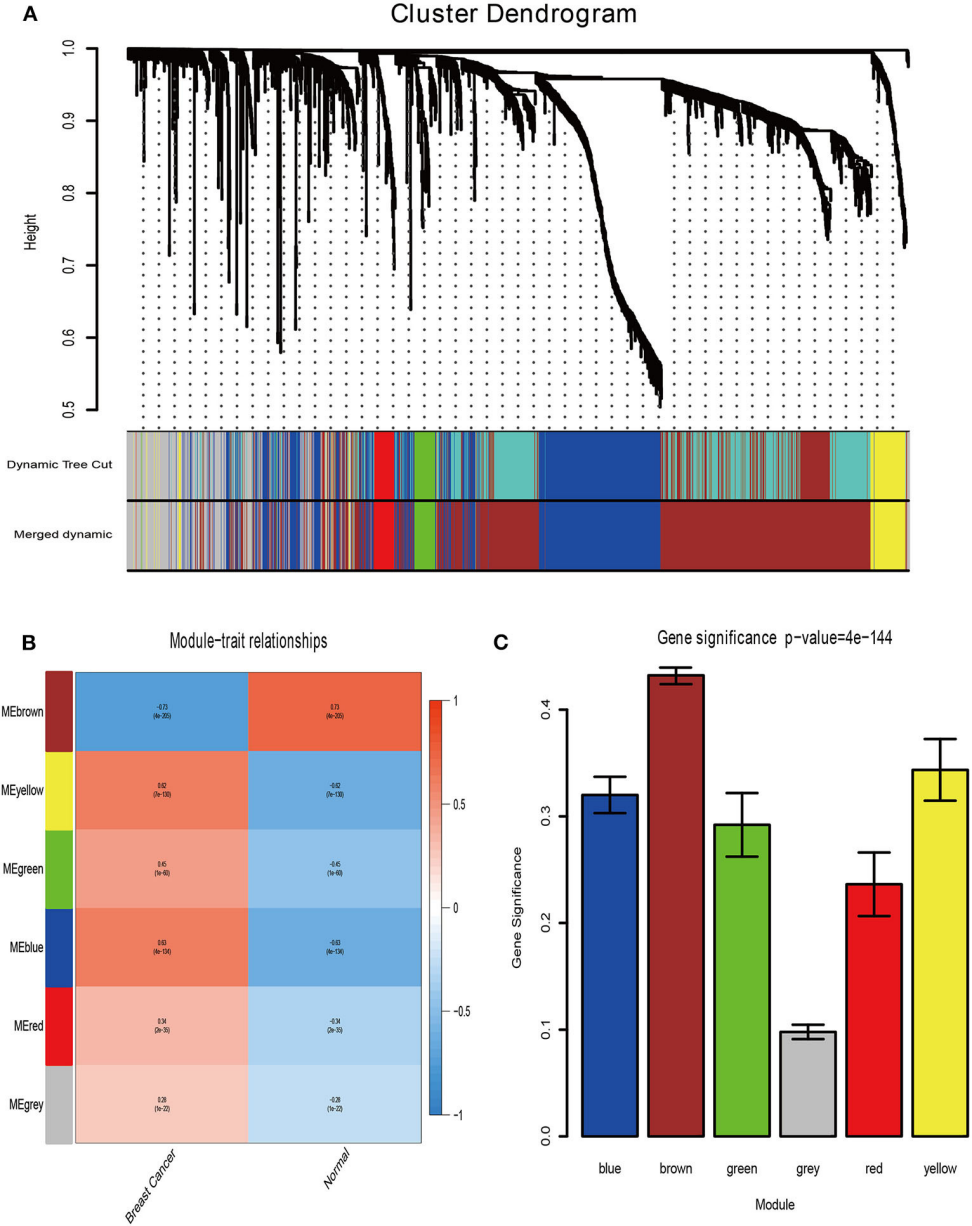
Determination of modules associated with clinical traits. **(A)** Dendrogram of all differentially expressed genes clustered on a dissimilarity measure (1-TOM). **(B)** Heatmap of the correlation between clinical traits of BRCA and module eigengenes. **(C)** Distribution of the mean gene significance and errors in the modules associated with BRCA status.

Firstly, the genes for co-expression network analysis were selected by differential expression analysis in TCGA dataset. A total of 1844 DEGs (1141 up-regulated and 703 down-regulated) were identified using TCGA dataset (Figure 3A). Meanwhile, 1106 DEGs (707 up-regulated and 399 down-regulated) were identified in BD samples vs. model samples in GSE85871 (Figure 3B). Then, the 1844 DEGs were analyzed for co-expression modules by the average linking clustering implemented in WGCNA package. In this work, the power of β = 6 (scale free R_2_ = 0.9) was chosen to ensure a scale-free network (Figures 4A–D). Then, the hierarchical clustering tree was determined by conducting hierarchical clustering for dissTOM and 6 modules were determined (Figure 5A). Subsequently, two methods were used to test the relations between each module and disease condition. Modules with a larger MS were considered to have more relations with disease condition. We found that the ME in brown module displayed a highest correlation with disease condition (Figure 5B). In addition, the ME of the brown module also showed the highest gene significance (Figure 5C). Finally, the brown module was selected for further analysis.

### Identification of Key Pathways and Key Genes

**Figure 6:**
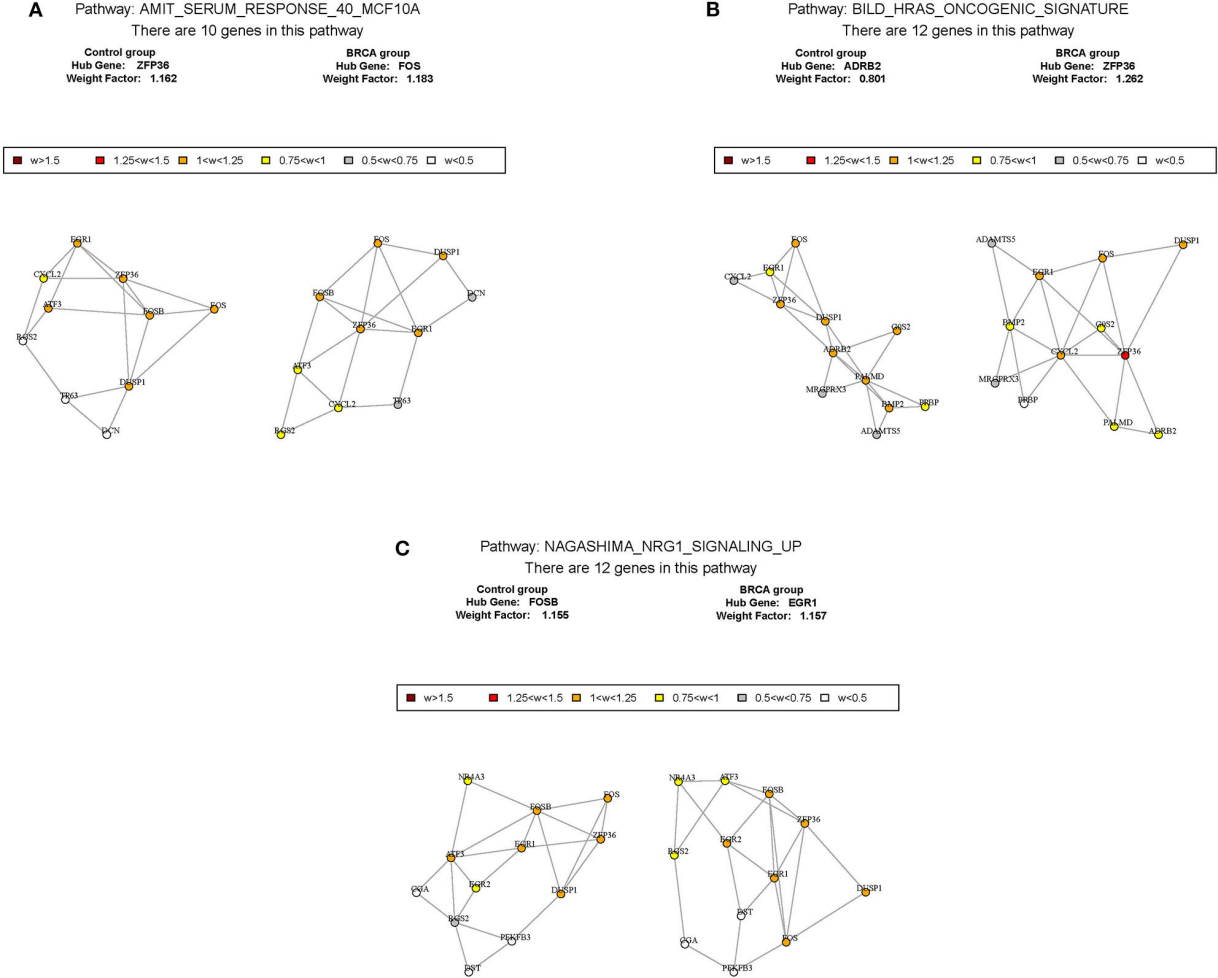
The construction of correlation network. The package GSAR was used to clarify the associated pathway and their key genes in control samples and BRCA samples. Three biological pathways associated with BRCA status were screened by GSNCA **(A–C)**.

**Figure 7:**
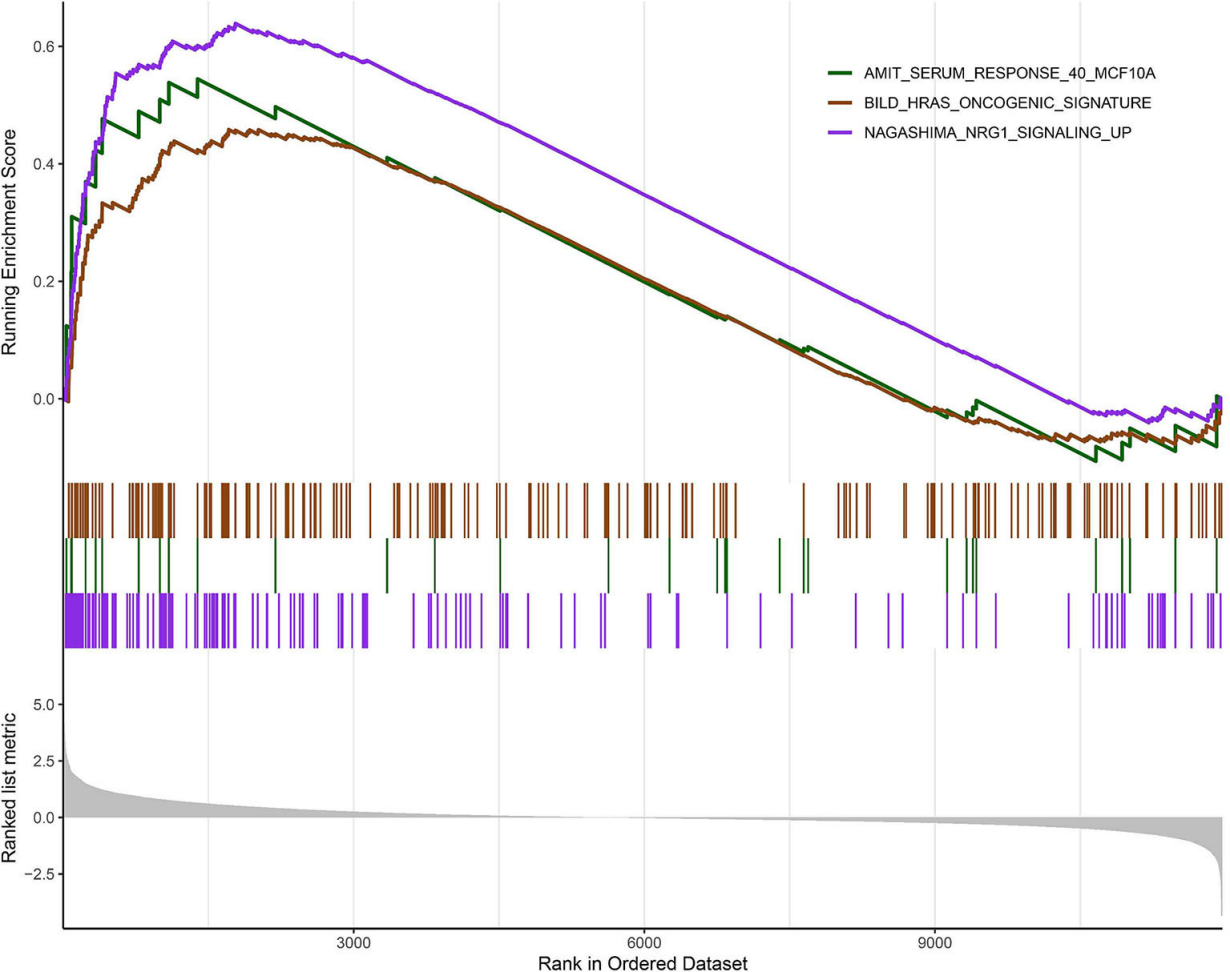
Gene set enrichment analysis (GSEA). The graph depicts the three gene sets from MSigDB enriched in BD samples.

**Figure 8:**
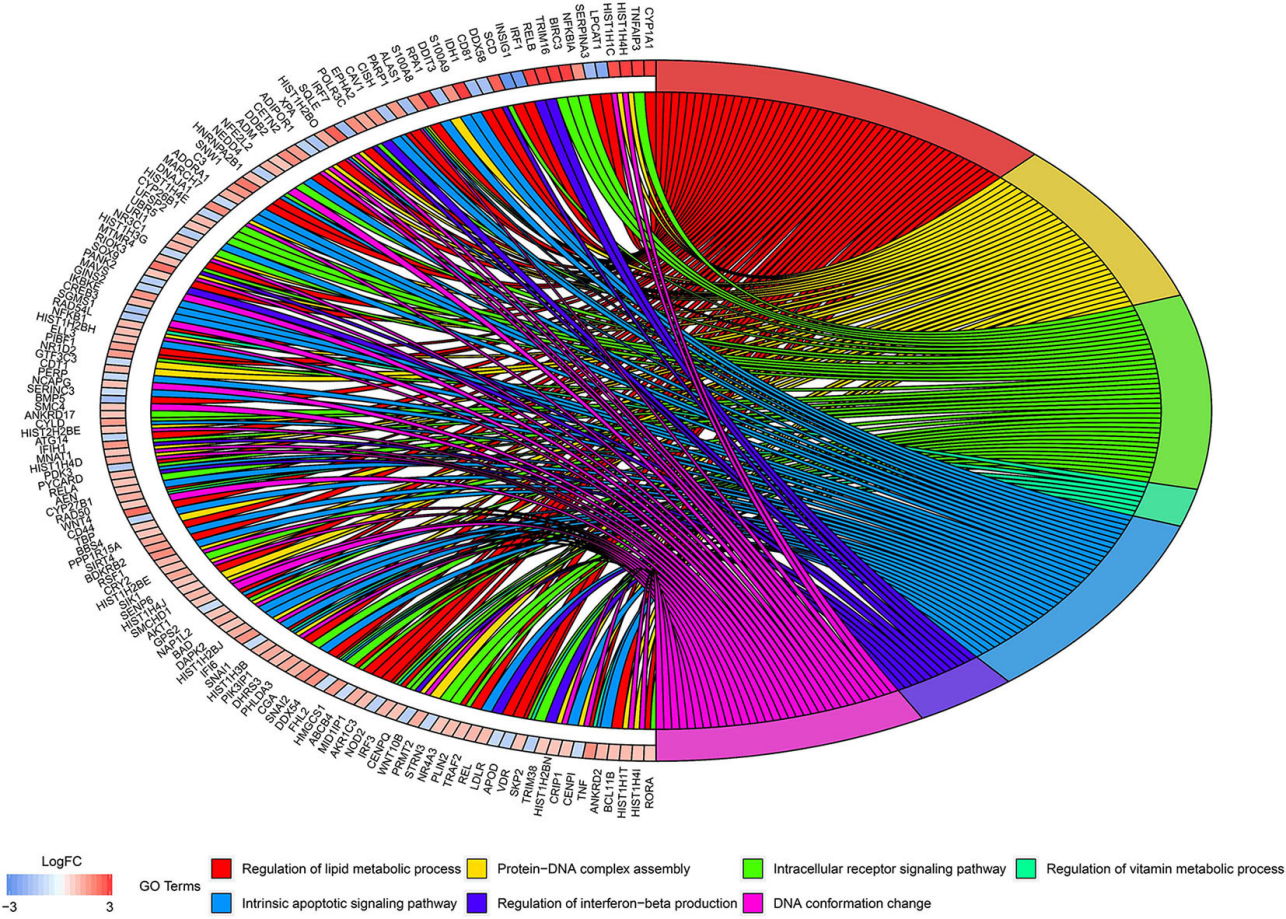
Circos plots of closely related GO terms and differentially expressed genes belonging to relevant GO terms. Gene involvement in the GO terms was identified by colored connecting lines.

According to the above analysis results, the hub module was selected to identify the key pathways and key genes. Finally, the AMIT_SERUM_RESPONSE_40_MCF10A, BILD_HRAS_ONCOGENIC_SIGNATURE, and NAGASHIMA_NRG1_SIGNALING_UP pathways were identified and ZFP36, EGR1, and FOS genes were identified as key genes by MST2 plot in BRCA samples (Figures 6A–C). Simultaneously, we also performed GSEA to identify the potential therapeutic mechanism of BD in GSE85871 dataset. Interesting, the pathways were also significantly enriched (Figure 7). In addition, the w-values of three key genes above are increased in BRCA samples, indicating their regulatory roles in breast cancer samples and their loss in the control samples (21). Although the w-values of the three genes was decreased in control samples, these genes were still close to each other in structure of MST2 in control samples, this suggests that correlation of them with other genes was not completely lost (21). Additionally, to further find the intervention biological processes of BD, we also performed functional enrichment analysis. The GO terms of biological process were most significantly enriched in regulation of lipid metabolic process, protein-DNA complex assembly, intracellular receptor signaling pathway, intrinsic apoptotic signaling pathway, regulation of interferon-beta production and DNA conformation change (Figure 8). Detail parameters of pathways and GO terms are shown in Tables 1–3. These results suggest the BD may affect these pathways and biological process and thus inhibited breast cancer cells proliferation and induced their apoptosis.

**Table 1:** Identification of crucial genes and pathways in BRCA samples by co-expression analysis.

**Pathway**	**GSNCA_pvalue**	**Genes**	**Count**
AMIT_SERUM_RESPONSE_40_MCF10A	0.0210	ATF3 TP63 CXCL2 EGR1 DUSP1 DCN FOS ZFP36 FOSB RGS2	10
BILD_HRAS_ONCOGENIC_SIGNATURE	0.0478	PALMD G0S2 PPBP CXCL2 EGR1 ADRB2 DUSP1 MRGPRX3 FOS BMP2 ZFP36 ADAMTS5	12
NAGASHIMA_NR[[Mathtype-mtef1-eqn-1.mtf]] G1_SIGNALING_UP	0.0100	NR4A3 RGS2 ATF3, FOSB, ZFP36, EGR2, EGR1, DUSP1, DST, FOS, CGA, PFKFB3	12

**Table 2:** Gene set enrichment analysis of differently expressed genes in BD samples.

**Description**	**enrichmentScore**	**NES**	***p-*value**
NAGASHIMA_NRG1_SIGNALING_UP	0.6390	2.2957	0.0013
AMIT_SERUM_RESPONSE_40_MCF10A	0.5445	1.5103	0.0246
BILD_HRAS_ONCOGENIC_SIGNATURE	0.4584	1.6816	0.0012

**Table 3:** GO enrichment analysis of differently expressed genes in BD samples.

**ID**	**Description**	**GeneRatio**	***p*-value**
GO:0019216	cellular response to external stimulus	0.0432	<0.001
GO:0065004	protein-DNA complex assembly	0.0319	<0.001
GO:0030522	intracellular receptor signaling pathway	0.0370	<0.001
GO:0030656	regulation of vitamin metabolic process	0.0007	<0.001
GO:0097193	intrinsic apoptotic signaling pathway	0.0360	<0.001
GO:0032648	regulation of interferon-beta production	0.0356	<0.001
GO:0071103	DNA conformation change	0.0329	<0.001

### Validation of Key Genes by qRT-PCR and Western Blot Assays

**Figure 9:**
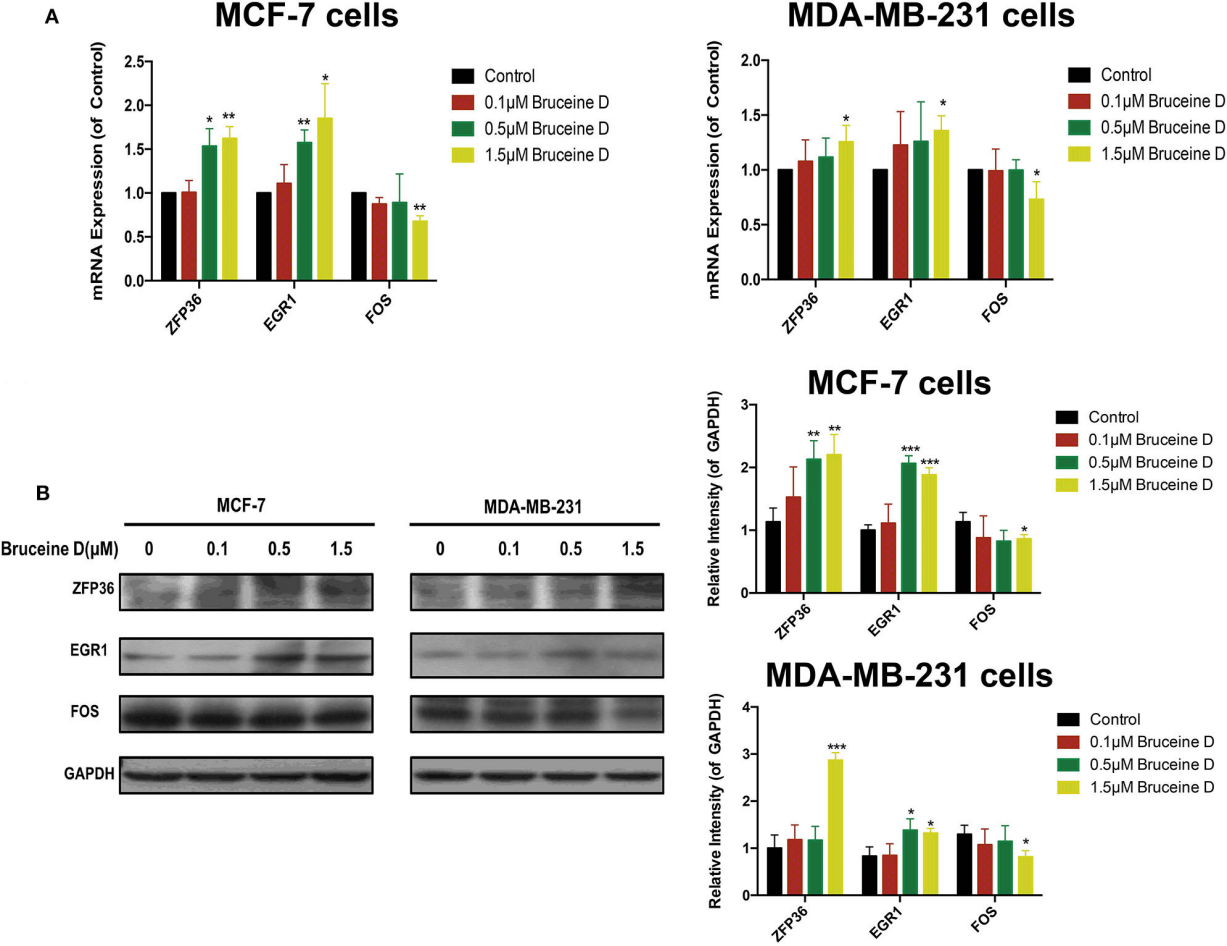
Validation of Key Genes by qRT-PCR and Western Blot. **(A)** The effects of BD on ZFP36, EGR1, and FOS mRNA expression in MCF-7 and MDA-MB-231 breast cancer cells were analyzed by qRT-PCR. **(B)** The effects of BD on FOS, ZFP36, and EGR1 protein expression in MCF-7 and MDA-MB-231 breast cancer cells were detected by western blot. *p < 0.05 to control group; **p < 0.01 to control group; ***p < 0.001 to control group (0 μM).

To validate the effects of BD on key genes, we initially quantified its effect on the level of ZFP36, EGR1 and FOS genes mRNA. As shown in Figure 9A, at three concentration of BD, 0.1, 0.5, and 1.5 μM, the expression of ZFP36 and EGR1 mRNA were increased in MCF-7 and MDA-MB-231 cells in a dose-dependent manner, while the level of FOS mRNA was significantly reduced in a concentration-dependent manner in MCF-7 and MDA-MB-231 breast cancer cells. We then determined whether BD was able to regulate ZFP36, EGR1, and FOS protein expression by western blot assays (Figure 9B). In the 0.5 and 1.5 μM BD-treated MCF-7 breast cancer cells, the expression levels of ZFP36 and EGR1 were markedly increased compared with DMSO-treated. In addition, the treatment of 1.5 μM BD significantly decreased the expression of FOS in MCF-7 cells. In MDA-MB-231 cells, 1.5 μM BD displayed a significant increase in the expression of ZFP36 and EGR1, and a marked inhibition in FOS expression. However, the treatment of 0.5 μM BD only significantly promoted EGR1 expression in MDA-MB-231 cells. These results indicated that BD could induce S phase cell cycle arrest and apoptosis in MCF-7 and MDA-MB-231 cells probably by promoting the expression of ZFP36 and EGR1 and inhibiting the expression of FOS.

## Discussion

Bruceine D, a tetracyclic triterpene quassinoid, is one of the active compounds of the Chinese herb *Brucea javanica*. Several studies have shown that BD exhibits good pharmacological activity including anti-inflammatory (23), anti-parasitic (24), hypoglycemic activities (25), and anti-tumor activity such as hepatocellular carcinoma (2), human chronic myeloid leukemia (26), and pancreatic cancer (27). However, by literature search, we found that therapeutic effects and mechanisms of BD have been rarely reported in breast cancer. In this study, we firstly demonstrated that BD significantly inhibited MCF-7 and MDA-MB-231 proliferation and induced S phase cell cycle arrest and apoptosis, which indicates BD could exhibit anti-breast cancer pharmacological activity. Then, we utilized computational analysis to investigate the mechanism of BD intervention in breast cancer. We built a weighted co-expression network and identified six modules from BRCA. And the brown module showed the best correlation with BRCA by correlation analyses. In addition, GSNCA and GSEA was conducted to identify key pathways and key genes to elucidate molecular mechanisms of BD. The results showed BD in breast cancer treatment may take actions through affecting AMIT_SERUM_RESPONSE_40_MCF10A, BILD_HRAS_ONCOGENIC_SIGNATURE, and NAGASHIMA_NRG1_SIGNALING_UP pathways and regulation expression of ZFP36, EGR1, and FOS(c-FOS). More importantly, the key pathways are closely associated with cancer. For example, it was reported that a number of genes within AMIT_SERUM_RESPONSE_40_MCF10A pathway are down-regulated in diverse tumor types and have a correlation with clinical outcomes. BILD_HRAS_ONCOGENIC_SIGNATURE pathway can discriminate cells expressing activated HRAS from control cells expressing GFP and distinguish between specific cancers and tumor subtypes. By making use of these oncogenic pathway signatures, it provides an opportunity to guide the use of targeted therapeutics. It has been confirmed that NAGASHIMA_NRG1_SIGNALING_UP pathway was associated with dose-dependent transient and sustained intracellular signaling, proliferation and differentiation of breast cancer cells.

Additionally, some studies have revealed three key genes play an important role in cancer development. ZFP36 is a tandem zinc finger protein in TTP family, which can directly bind AU-rich elements (ARE) and destabilize the host transcript. Several studies showed that ZFP36 binds to and degrades the GM-CSF, TNFα, and VEGF mRNAs, in turn affecting the function of transcription factors such as NF-κB, which has a vital impact on cell viability (28, 29). In breast cancer, ZFP36 gene defects will contribute to cancer progression and development. Our results showed that BD could significantly increase ZFP36 mRNA and protein expression in a dose-dependent manner in MCF-7 and MDA-MB-231 breast cancer cells. EGR1 is a tumor suppressor and an oncogene (30–32). As a transcription factor, EGFR can active or repress gene transcription (33, 34). In addition, the transcription of EGR1-mediated brings together a multitude of signaling cascades, which have a vital impact on cell growth, apoptosis and differentiation (33, 35). Recent studies have demonstrated the in breast cancer EGR1 defects are associated with poor prognosis, which is independent of subtype (36). In accordance with this context, we illustrated that EGR1 may be a BD's target for breast cancer treatment. FOS (c-FOS) is one of FOS family protein, which contains transcription factors such as Fra-1, Fra-2, FosB, FosB2, and ΔFosB (37). FOS plays a relevant role in the regulation of normal cell growth, differentiation, and cellular transformation processes (38, 39). The *c-Jun* family members can interact with nuclear protein, which was encoded by FOS, to form the heterodimeric activating protein-1 (AP-1) transcription factor complex (40). This complex binds to DNA at its specific sites in promoters and enhancer regions and causes changes in gene expression patterns, which might be critical for cell cycle progression. Normally, FOS protein plays a major role in several cellular functions, and its overexpression has been observed in many neoplasias (41). In the present study, we demonstrated that BD significantly decreased the level of FOS mRNA and protein expression in MCF-7 and MDA-MB-231 breast cancer cells. Hence, these key genes are likely to be potential targets of BD. However, there were some limitations in our study. Above all, in our paper, we just chose differentially expressed genes for weighted correlation network analysis. This did not include all the genes that may be associated with breast cancer. Then, there was only two biological replicates in GSE85871 dataset, which may affect the accuracy of the results. Finally, experimental studies need to be carried out to verify the three potential targets of BD in future work.

In summary, by integrated multiple computation methods, we found that the BD may influence some key pathways that are closely associated with cancer, such as AMIT_SERUM_RESPONSE_40_MCF10A, BILD_HRAS_ONCOGENIC_SIGNATURE, and NAGASHIMA_NRG1_SIGNALING_UP pathways. Simultaneously, the key genes, including ZFP36, EGR1, and FOS may be potentially effective targets of BD and also are crucial for breast cancer development. Additionally, this is the first work to report therapeutic effects and possible molecular mechanisms of BD in breast cancer which would future improve our understanding of BD.

## Data Availability Statement

Publicly available datasets were analyzed in this study, these can be found in The Cancer Genome Atlas (https://portal.gdc.cancer.gov/) (TCGA-BRCA); the NCBI Gene Expression Omnibus (GSE85871).

## Author Contributions

ST and RJ collected and analyzed the data, drafted, and revised the manuscript. WZ designed the study and revised the manuscript. All authors contributed to the article and approved the submitted version.

## Conflict of Interest

The authors declare that the research was conducted in the absence of any commercial or financial relationships that could be construed as a potential conflict of interest.
